# Influence of sire and maternal grandsire breed on functional and production traits in organic dairy cows

**DOI:** 10.3168/jdsc.2025-0969

**Published:** 2026-03-06

**Authors:** T. Muratori, B. Heins, L. Hardie, B. Basiel, C. Dechow

**Affiliations:** 1 Department of Animal Science, Pennsylvania State University, University Park, PA 16802; 2 Department of Animal Science, University of Minnesota, St. Paul, MN 55108; 3 ABS Global Inc., DeForest, WI 53532; 4 Department of Animal Science, University of Vermont, Burlington, VT 05405

## Abstract

This study evaluated the effects of sire and maternal grandsire (MGS) breed on functional and production traits of 3,623 lactating dairy cows across 14 certified organic US herds and examined phenotypic associations between these traits and test-day milk performance. Data were analyzed using linear mixed models to compare Holstein (HO), Jersey (JE), Montbéliarde (MO), Red Dairy Cattle (RDC), and other (OT) breed. The HO-sired cows and HO-MGS cows had the greatest milk yield but only marginal advantages in fat and protein yields compared with other breeds. The HO and OT-sired cows exhibited higher fly scores and fly counts. Also, the HO-sired cows had the poorest hygiene among the breeds studied. The MO, and RDC-sired cows had greater BCS and JE cows were shorter and had udder floors closer to their hock. The MGS effects followed similar but weaker trends as the sire breed, with MO and RDC associated with higher BCS and improved hygiene. Regression coefficients show that reduced milk, protein and fat yield were linked to higher BCS, poorer locomotion, and shallow udders, whereas cows with a taller stature had increased yields. The results of this study reveal breed-based differences for production and functional traits in a population of dairy cows housed in an organic and pasture-based production system. This study emphasizes the potential for utilizing genetic differences between breeds to optimize production and functional traits of organic dairy cows and to enhance the sustainability of organic dairy farming.

## Highlights

• Holstein-sired cows had the highest yield but poorer hygiene and body condition.
• Montb-liarde- and Red Dairy Cattle-sired cows were cleaner and maintained better BCS.
• Fly scores were highest in Holstein- and other-sired cows on pasture.
• Higher BCS and shallow udders were negatively associated with yield.
• Structural traits should guide sire selection for organic dairy systems.

## Graphical Abstract

This study evaluated how sire and maternal grandsire breed groups influence functional and production traits in organic dairy cows under pasture-based management. Using data from 3,623 cows across 14 certified organic farms, results showed significant breed-based differences in fly burden, body condition, stature, udder depth, and hygiene, and their associations with milk yield and composition. Holstein-sired cows exhibited the highest milk yield but also the highest fly loads, lowest body condition score (BCS), shallower udders, and poorer hygiene. In contrast, Montb-liarde and Red Dairy Cattle genetics were associated with better body condition and cleanliness, with only modest reductions in yield. Regression analyses indicated that higher body condition and shallow udders were negatively associated with milk production, and stature was positively linked to yield. These findings highlight the importance of integrating structural and welfare traits into breeding decisions to enhance productivity and sustainability in organic, low-input dairy systems.

**Figure:**
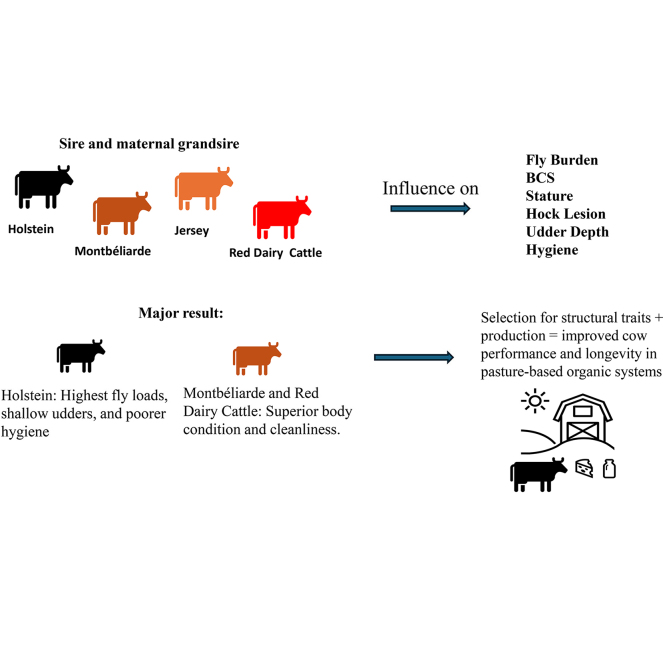

Organic dairy production in the United States is governed by a defined set of rules, which brings both benefits and challenges. Using breeds that thrive in organic production systems may be a solution to the challenges that face organic cows. For example, pasture must comprise at least 30% of DMI during the grazing season (Moscovici Joubran et al., 2021), and antibiotics and pesticides are prohibited (Brock et al., 2021; Schell et al., 2023). Hence, organic dairy farming can challenge the cow's energy balance, especially when organic supplements are costly or limited (Dechow et al., 2011; Hardie et al., 2014; Phillips and Heins, 2022). Organic dairy cows thus often have lower BCS, which can lead to poorer fertility and reduced disease resistance (Hardie et al., 2014; Gillespie, 2023). To maintain the production and health of cows in organic dairy systems, common preventive strategies are accomplished through optimizing nutrition or husbandry (Kienitz et al., 2018; Sorge et al., 2016). Genetics and breeding, on the other hand, have a great and untapped potential to make organic dairy production more robust and sustainable.

Cows with Montbéliarde (**MO**), Jersey (**JE**), and Viking Red (**VR**) genetics, incorporated in structured crossbreeding programs, enhance reproductive performance, udder health, and component yields under grazing, compared with Holsteins (**HO**; Heins et al., 2006; Hazel et al., 2017; Liedgren et al., 2024), who have lower BCS and greater health issues in these systems (Washburn and Mullen, 2014). Lower BCS is associated with reduced fertility and altered milk composition (Buckley et al., 2003). Poor hock condition and locomotion impair grazing, limiting intake and decreasing milk and component yields (Mattachini et al., 2020). Deep udders increase mastitis risk and reduce milk quality (Gutiérrez Reinoso et al., 2023), and poor hygiene exacerbates udder health issues (Hazel et al., 2017). However, the effect of these traits on production across genetics in organic systems remains underexplored.

Fly burden has recently gained attention as a key phenotype in organic herds. Horn and stable flies impair behavior, udder health, and yield (Kienitz et al., 2018; Renčínová et al., 2021). In Holsteins, horn fly resistance showed moderate heritability, and selection for yield genetically increased fly load, which phenotypically reduced production (Basiel et al., 2021). However, variation across breeds remains underexplored.

This study aimed to investigate the differences in functional and production traits between different sire and maternal grandsire (**MGS**) breeds. Second, the study aimed to assess the associations between functional and production traits for the different breeds.

Data were collected from Summer 2017 to Summer 2019 across 14 organic US dairy herds, consisting of 3,623 lactating dairy cows from 5 sire breeds (**SB**): HO (n = 1,734), JE (n = 144), MO (n = 183), Red Dairy Cattle (**RDC**; n = 508), and other or unknown breeds (**OT**; n = 1,054). Herd sizes ranged from 87 to 1,184 cows. Holstein- and OT-sired cows were present in all 14 herds, MO- and JE-sired cows were each represented in 4 herds, and RDC-sired cows were represented in 7 herds. Maternal grandsire (**MGS**) breed distribution followed a similar pattern, with HO-MGS present in 12 herds, MO-MGS represented in 3 herds, JE-MGS represented in 7 herds, RDC-MGS represented in 6 herds, and OT-MGS represented in all 14 herds. The RDC group represented lactating cows sired by Norwegian Red, Swedish Red, Ayrshire, and Danish Red bulls. The OT breed incorporated lactating cows sired by Normande (n = 27), Milking Shorthorn (n = 24), Guernsey (n = 8), Brown Swiss (n = 16), Angus (n = 1), and unknown (n = 978) sires. The same breed classification was applied for the MGS groups. Sire breed × MGS combinations included HO × HO (n = 909), HO × OT (n = 688), HO × RDC (n = 80), MO × HO (n = 84), OT × HO (n = 70), OT × OT (n = 935), RDC × HO (n = 74), RDC × OT (n = 320), and other combinations of <70 cows.

All functional traits were scored by trained research personnel upon visual inspection. A total of 8 primary scorers contributed to the data collection, with 5 of the 8 scorers recording data in multiple herds. The BCS was scored from 1 to 5, where 1 was thin and 5 was fat (Wildman et al., 1982). Locomotion was scored from 1 (sound) to 5 (severely lame) based on the standard welfare assessment guidelines of the International Committee for Animal Recording (2020). Hock lesions was scored as follows: 0 = no injury, 1 = hair loss, 2 = swelling or scab, and 3 = severe swelling or open wound (Professional Animal Auditor Certification Organization, 2023). Hygiene was scored as follows: 1 = clean, 2 = light contamination, 3 = moderate contamination, and 4 = heavy contamination (Reneau et al., 2005). Fly scores was assigned on a scale from 0 to 4, where 0 indicated no flies and 4 indicated heavy infestation (Basiel et al., 2021); a photograph was taken from one side of each cow, and flies were manually counted from the image to derive fly count (Basiel et al., 2021). Stature and udder depth were visually appraised and converted to centimeters. Multiple farm visits yielded a functional and conformation dataset of 4,574 records from the 3,623 cows with SB of HO (n = 2,195), JE (n = 165), MO (n = 243), RDC (n = 567), and OT (n = 1,404). Functional and conformation traits included BCS (n = 3,900), fly score (n = 4,574), image-derived fly count (n = 719), stature (cm; n = 4,060), udder depth (cm from hock to udder floor; n = 3,879), locomotion (n = 3,965), hock lesion (n = 4,041), and hygiene score (n = 4,034). Not all traits were scored on every cow because of movement during pasture assessment; imaging was limited to a subset.

All test-date DHI records were collected through herd management software. The production traits collected included milk yield (n = 7,360), fat and protein content (n = 3,073), and SCS (n = 3,001) obtained for test days occurring within ± 30 days of scoring date. When an individual cow observation fell within both test-day windows, both test days were retained for analysis.

All statistical analyses were conducted using SAS (v9.4, SAS Institute Inc., Cary, NC), with significance declared at *P* ≤ 0.05 for all analyses. A series of linear mixed-effects models were fitted using the HPMIXED procedure in SAS according to the following model:

y_ijslmnpk_ = µ + SB_i_ + MGSB_j_ + Scorer_s_ + Farm_l_ + b_1_ × DIM + b_2_ × DIM^2^ + Lact_m_ + HYScalv_n_ + Date_p_ × Farm_l_ + C_k_ + ε_ijslmnpk_,

where y_ijslmnpk_ = fly score, fly count, BCS, stature, locomotion score, hock lesion score, hygiene score, udder depth, milk yield (kg), fat (kg and %), protein (kg and %), or SCS; SB_i_ = fixed effect of cow SB (i = HO, JE, MO, RDC, OT); MGSB_j_ = fixed effect of cow maternal grandsire breed (**MGSB**; j = HO, JE, MO, RDC, OT); Scorer_s_ = the fixed effect of the individual scoring the animal (s = 8); Farm_l_ = the fixed effect of the farm (l); b_1_ and b_2_ are regression coefficients for DIM and DIM^2^, respectively; Lact_m_ = the fixed effect of parity (m = 1, 2, ≥3); HYScalv_n_ = fixed effect of herd-year-season of calving (n); C_k_ = random effect of cow k; Date_p_ × Farm_l_ = random effect of date of collection p × farm l; and ε_ijslmnpk_ = residual error. Least squares means (LSM) were computed for SB, MGSB, and parity, and only retained when statistically significant. A random interaction between SB and MGSB was tested for stature and hygiene but not retained for other traits because it was not significant. Although DIM^2^ was retained only for BCS and production traits, DIM was included for fly score, fly count, stature, hock lesion score, hygiene score, and udder depth. A second model regressed test-day production traits (milk, protein and fat [yield and %], and SCS) on individual functional traits to assess their phenotypic associations using the HPMIXED procedure in SAS according to the following model:

y_gijklmnp_ = FT_g_ + SB_i_ + MGSB_j_ + b_1_ × DIM + b_2_ × DIM^2^ + Lact_m_ + HYScalv_n_ + Date_p_ × Farm_l_ + SB_i_ × MGSB_j_ + C_k_ + ε_gijklmnp_,

where y_gijklmnp_ = milk yield (kg), fat (kg and %), protein (kg and %), or SCS; FT_g_ = fly score, fly count, BCS, stature, locomotion score, hock lesion score, hygiene score, or udder depth; g = specific combination of date and farm of data collection; SB_i_ = fixed effect of cow SB (i = HO, JE, MO, RDC, OT); MGSB_j_ = fixed effect of cow MGSB (j = HO, JE, MO, RDC, OT); b_1_ and b_2_ = regression coefficients for DIM and DIM^2^, respectively; Lact_m_ = the fixed effect of parity (m = 1, 2, ≥3); HYScalv_n_ = fixed effect of herd-year-season of calving; n = specific combination of herd-year-season of calving; Date_p_ × Farm_l_ = random effect of date of collection p × farm l; SB_i_ × MGSB_j_ = random effect of SB i × MGSB j; C_k_ = random effect of cow k; and ε_gijklmnp_ = residual error.

Table 1 presents the association of SB with differences in both visually scored and image-derived fly counts. The HO- and OT-sired groups had the highest fly loads (1.03 and 0.96, respectively; *P* < 0.05), and HO-sire groups had the highest image-derived fly counts (33.13). Fly score was strongly correlated with image-derived fly counts (ρ = 0.75; *P* < 0.0001). The elevated fly scores and counts in HO mirror early studies that fly burden is heritable and has moderately positive genetic correlations with yield traits, suggesting that cows genetically predisposed for higher production may also be predisposed to host more flies. Basiel et al. (2021) reported moderate heritability for horn fly resistance (0.25 ± 0.04) in organic HO, indicating substantial within-breed variation, and noted lower fly loads in cattle with greater white coat coloration.

**Table 1:** Means ± SD for functional traits by sire breed (SB) and maternal grandsire breed (MGSB) in organic dairy cows

Trait	Scoring unit	SB					MGSB				
		HO	JE	MO	OT	RDC	HO	JE	MO	OT	RDC
Fly score	0–4	1.03 ± 0.09^a^	0.80 ± 0.11^b^	0.80 ± 0.10^b^	0.96 ± 0.09^c^	0.87 ± 0.09^b^	0.90 ± 0.08	0.94 ± 0.10	0.78 ± 0.11	0.89 ± 0.08	0.94 ± 0.10
Fly count	n	33.13 ± 11.14^a^	13.59 ± 13.13^b^	3.36 ± 13.33^b^	32.34 ± 11.30^ab^	14.92 ± 12.22^b^	22.70 ± 10.99^a^	28.16 ± 12.79^a^	15.53 ± 14.95^ab^	25.37 ± 10.96^a^	5.58 ± 12.82^b^
BCS	1–5	2.96 ± 0.04^a^	2.95 ± 0.06^ad^	3.50 ± 0.06^b^	3.06 ± 0.44^d^	3.21 ± 0.05^c^	3.06 ± 0.04^a^	3.08 ± 0.06^ac^	3.28 ± 0.07^b^	3.12 ± 0.04^c^	3.16 ± 0.05^c^
Stature	cm	141.94 ± 0.54^a^	136.86 ± 0.69^b^	140.84 ± 0.64^a^	140.95 ± 0.60^a^	140.32 ± 0.55^c^	141.22 ± 0.53^a^	137.93 ± 0.65^b^	140.49 ± 0.72^a^	141.17 ± 0.52^a^	140.11 ± 0.63^a^
Locomotion	1–5	3.05 ± 0.12	2.88 ± 0.15	3.10 ± 0.14	3.05 ± 0.12	2.99 ± 0.12	3.05 ± 0.12	2.95 ± 0.15	3.02 ± 0.16	3.01 ± 0.11	3.04 ± 0.14
Hock lesion	0–3	0.13 ± 0.05	0.11 ± 0.06	0.157 ± 0.06	0.14 ± 0.05	0.14 ± 0.05	0.15 ± 0.05	0.10 ± 0.06	0.12 ± 0.07	0.16 ± 0.05	0.14 ± 0.05
UD^1^	cm	8.56 ± 0.36^a^	5.70 ± 0.54^b^	5.99 ± 0.49^b^	7.85 ± 0.37^c^	6.88 ± 0.38^d^	7.27 ± 0.36^a^	6.70 ± 0.53^ab^	7.41 ± 0.56^ab^	6.80 ± 0.35^b^	6.81 ± 0.47^ab^
Hygiene	0–4	1.59 ± 0.09^a^	1.47 ± 0.12^ab^	1.46 ± 0.11^ab^	1.41 ± 0.10^b^	1.39 ± 0.10^b^	1.58 ± 0.09^a^	1.44 ± 0.12^ab^	1.34 ± 0.12^b^	1.52 ± 0.09^ab^	1.43 ± 0.11^ab^

a–d Different superscript letters within rows indicate differences between groups at *P* < 0.05.

1 UD = udder depth.

The MO-sired cows exhibited the greatest BCS (3.50), followed by RDC (3.21), contrasting with significantly lower values in HO (2.96) and JE (2.95; *P* < 0.05). This agrees with Hazel et al. (2017), whose crossbreeding study showed that MO × HO and VR × HO crosses maintain higher BCS in first lactation than pure HO. Because BCS is tied to reproductive performance, these higher values may suggest enhanced fertility potential under organic pasture-based management. No significant SB differences were seen for locomotion or hock lesions.

The HO-sired cows had significantly higher udder floors from the hocks (8.56 cm) than JE (5.70 cm) and MO (5.99 cm; *P* < 0.05). Deeper udders, although often linked to greater milk capacity, may increase mastitis risk under pasture conditions (Pantoja et al., 2016). Cows sired by HO had significantly higher hygiene scores (1.59) than RDC (1.39) and OT (1.41; *P* < 0.05).

Table 2 shows that HO-sired cows had the greatest milk yield (29.17 kg), exceeding JE (25.81 kg; *P* < 0.05), with MO, RDC, and OT groups being intermediate. Differences in fat percentage were not significant (3.89% to 4.01%), although fat yield was greater for HO (0.99 kg) than OT (0.94 kg; *P* < 0.05). Protein yield followed a similar pattern (HO at 0.76 kg vs. OT at 0.73 kg; *P* < 0.05), while SCS did not differ across sire groups (3.31 to 3.94). These findings align with data from Heins et al. (2008), who reported that JE × HO crossbreds produced significantly less milk (7,147 kg) than pure HO (7,705 kg; *P* < 0.01), but fat yield (274 vs. 277 kg; *P* > 0.05), protein yield (223 vs. 238 kg; *P* < 0.01), and SCS (3.22 vs. 2.95; *P* > 0.05) showed similar trends, confirming the superior milk volume but comparable milk-composite yield of HO (Heins et al., 2008).

**Table 2:** Means ± SD for production traits by sire breed (SB) and maternal grandsire breed (MGSB) in organic dairy cows

Trait	Unit	SB					MGSB				
		HO	JE	MO	OT	RDC	HO	JE	MO	OT	RDC
Milk yield	kg	29.17 ± 1.29^a^	25.81 ± 1.56^b^	28.17 ± 1.46^ab^	27.62 ± 1.37^ab^	28.10 ± 1.32^ab^	28.88 ± 1.27^a^	26.41 ± 1.56^b^	27.58 ± 1.61^ab^	28.37 ± 1.25^ab^	27.65 ± 1.45^ab^
Fat	%	3.89 ± 0.10	4.01 ± 0.16	3.84 ± 0.16	3.91 ± 0.11	3.92 ± 0.15	3.86 ± 0.10^a^	4.09 ± 0.16^ab^	3.98 ± 0.18^ab^	3.94 ± 0.10^b^	3.70 ± 0.18^ab^
Fat yield	kg	0.99 ± 0.04^a^	0.95 ± 0.06^ab^	0.97 ± 0.06^ab^	0.94 ± 0.04^b^	0.98 ± 0.06^ab^	0.98 ± 0.04	0.97 ± 0.07	0.97 ± 0.07	0.98 ± 0.04	0.92 ± 0.07
Protein	%	2.98 ± 0.05	3.09 ± 0.07	3.04 ± 0.07	3.02 ± 0.05	3.00 ± 0.07	2.98 ± 0.04^a^	3.13 ± 0.07^b^	3.02 ± 0.08^ab^	3.034 ± 0.05^b^	2.98 ± 0.08^ab^
Protein yield	kg	0.76 ± 0.03^a^	0.74 ± 0.04^ab^	0.77 ± 0.04^ab^	0.73 ± 0.03^b^	0.76 ± 0.04^ab^	0.78 ± 0.03	0.73 ± 0.04	0.75 ± 0.05	0.76 ± 0.04	0.74 ± 0.05
SCS		3.70 ± 0.30	3.94 ± 0.45	3.31 ± 0.47	3.62 ± 0.31	3.60 ± 0.44	3.48 ± 0.27	4.11 ± 0.47	3.60 ± 0.53	3.38 ± 0.28	3.60 ± 0.54

a,b Different superscript letters within rows indicate significant differences between groups at *P* < 0.05.

In general, MGS effects paralleled sire effects but were smaller. The HO-MGS cows had higher milk yield (28.88 kg) than JE-MGS (26.41 kg; *P* < 0.05). Fat percentage tended to be higher in JE (4.09%) and MO (3.98%) than HO (3.86%). Protein percentage was higher in JE (3.13%) than HO (2.98%; *P* < 0.05). The weaker MGS patterns reflect their smaller genomic contribution (25%).

Regression coefficients in Table 3 show weak associations between fly score and milk yield (β = −0.100; *P* < 0.43) and a small positive association with fat yield (β = 0.009; P < 0.01); image-derived fly counts were not associated with production. Previous research demonstrated that high-yielding cows appear genetically predisposed to host more flies, which masks the true effect of fly load on milk yield (Basiel et al., 2021); this could explain the relatively small relationship between fly score and milk yield here.

**Table 3:** Regression coefficients for functional traits on production traits in organic dairy cows

Trait	Scoring unit	Milk yield (kg)	Fat %	Fat yield (kg)	Protein %	Protein yield (kg)	SCS
Fly score	0–4	−0.100	0.013	0.009**	−0.004	0.002	0.029
Fly count	n	0.003	−0.001	0.001	−0.001	0.001	0.002
BCS	1–5	−1.145***	0.001	−0.024***	0.002***	−0.013***	0.004
Stature	cm	0.347***	−0.003	0.005**	−0.003	0.003**	0.008
Locomotion	1–5	−1.041***	0.003	−0.006	0.001	−0.011**	0.018
Hock lesion	0–3	0.351	−0.031*	−0.002	−0.027***	−0.006	−0.047
UD^1^	cm	−0.403***	−0.002	−0.007***	0.002	−0.003***	−0.018
Hygiene	0–3	0.711***	0.001	0.007*	−0.001	0.005*	0.011

1 UD = udder depth.

* *P* < 0.05, **P < 0.01, ***P < 0.001.

Regression analyses confirmed that higher BCS was associated with lower milk (β = −1.145; *P* < 0.001), fat (β = −0.024; *P* < 0.001), and protein yields (β = −0.013; *P* < 0.001), yet positively related to protein percentage (β = 0.002; *P* < 0.001), consistent with energy-reserve allocation trade-offs in high-yielding cows. Notably, our MO-sired cows maintained higher BCS under grazing conditions without a meaningful yield loss. This observation aligns with the work of Pires et al. (2015), who found higher postpartum BCS (1.43 vs. 1.12; *P* < 0.001), less condition loss (1.03 vs. 1.41 units; *P* = 0.001), and fewer ovarian abnormalities (32% vs. 58%; *P* < 0.10) in MO versus HO cows under low-input, spring-calving systems. Similarly, Hazel et al. (2017) reported that MO × HO crossbreds had a 0.50 ± 0.02 higher BCS (*P* < 0.001), a 7-percentage-point improvement in first-service conception rate, and a 10-d shorter interval to conception (*P* < 0.01), without a reduction in fat + protein yield. All these results support potential fertility, energy balance, and physiological resilience advantages of the MO breed in grazing and low-input systems.

Taller cows had higher milk (β = 0.347; *P* < 0.001), protein (β = 0.003; *P* < 0.01), and fat yields (β = 0.005; *P* < 0.01), consistent with Dechow et al. (2011), who suggested that larger-framed cows possess somewhat greater feed intake or production capacity.

Higher locomotion scores (poorer mobility) were negatively associated with milk (β = −1.041; *P* < 0.001) and protein yields (β = −0.011; *P* < 0.01). This is in line with the work of Green et al. (2002), who found that cows with clinical lameness experienced reduced yield starting up to 4 mo before diagnosis and continuing for 5 mo after, with a mean estimated loss of 360 kg per 305-d lactation. Similarly, Onyiro et al. (2008) showed that HO cows with a locomotion score of 4, had an average daily milk loss of 0.78 kg relative to sound cows. More recently in pasture-based systems, O'Connor et al. (2020) found that a mobility score of 3 (severely impaired) was associated with a yield reduction of 299 to 356 kg (305-d basis) compared with optimal mobility. In our study, cows with poorer mobility produced significantly less milk and protein, underscoring that locomotion is not only a welfare issue but also an economically relevant trait. Therefore, selecting breeds with better mobility could help sustain production in pasture-based organic systems where walking demands are high.

Cows with higher hock lesion scores produced milk with lower fat (β = −0.031; *P* < 0.05) and protein percentages (β = −0.027; *P* < 0.001), reflecting possible structural stress or metabolic trade-offs in higher-yielding cows. Furthermore, shallow udders were negatively associated with milk (β = −0.403; *P* < 0.001), fat (β = −0.007; *P* < 0.001), and protein yields (β = −0.003; *P* < 0.001).

Better hygiene was unfavorably associated with milk (β = 0.711; *P* < 0.001), fat (β = 0.007; *P* < 0.05), and protein yields (β = 0.005; *P* < 0.05), indicating that higher-producing cows tended to be less clean. Similarly, DeVries et al. (2012) reported that cows with greater milk yield exhibited poorer udder hygiene (β = 0.011; *P* = 0.007) and dirtier lower legs (β = 0.003; *P* = 0.03). They hypothesized that high-yielding cows may spend more time standing and feeding near manure-contaminated alleyways in confinement systems, thereby increasing their exposure to manure. However, due to our cows being on pasture for most of the day, a potential explanation is that high-yielding cows eat and defecate more frequently. Moreover, higher DMI was previously associated with looser fecal consistency (Ireland-Perry and Stallings, 1993). Collectively, these observations suggest that higher productivity may predispose cows to compromised hygiene, possibly due to behavioral and anatomical factors. (DeVries et al., 2012).

These results are particularly relevant to pasture-based organic systems, where limited inputs place greater emphasis on resilience and functional traits. Although some trends may hold in conventional settings, the benefits of robust body condition and reduced susceptibility to stressors may be more critical under low-input management, reinforcing the need for system-specific breeding strategies.

In conclusion, HO-sired cows had the greatest milk, fat, and protein yields but also exhibited higher fly loads, poorer hygiene, and lower BCS. The MO- and RDC-sired cows maintained superior body condition with only modest yield reductions, indicating potential reproductive and health advantages. Impaired mobility, shallow udders, poor hygiene, and hock lesions were all unfavorably associated with milk or component yields. These findings underscore the importance of selecting SB that combine production traits with functional traits to support performance as well as health and welfare of dairy cows in pasture-based organic dairy systems.
